# Effectiveness of Mental Health Apps for Distress During COVID-19 in US Unemployed and Essential Workers: Remote Pragmatic Randomized Clinical Trial

**DOI:** 10.2196/41689

**Published:** 2022-11-07

**Authors:** Katherine Anne Comtois, Felicia Mata-Greve, Morgan Johnson, Michael D Pullmann, Brittany Mosser, Patricia Arean

**Affiliations:** 1 Advanced Laboratories for Accelerating the Reach and Impact of Treatments for Youth and Adults with Mental Illness Department of Psychiatry and Behavioral Sciences University of Washington Seattle, WA United States; 2 Conducting Research to Enhance Assessment and Treatment Through Innovation in Mental Health Lab Department of Psychiatry and Behavioral Sciences University of Washington Seattle, WA United States

**Keywords:** COVID-19, COVID, coronavirus, pandemic, SARS-CoV-2, essential worker, suicide, suicidal, commercial app, mental health apps, health app, mental health, mHealth, mobile health, occupational health, employee, employment, unemployed, worker, job, depression, anxiety, stress, distress, mobile app, RCT, pragmatic trial, randomized, health care worker, health care provider, frontline staff

## Abstract

**Background:**

During the COVID-19 pandemic, the general public was concerned about the mental health impacts of unemployment due to COVID-19 and the stress essential workers experienced during this time. Several reports indicated that people in distress were turning to digital technology, but there was little evidence about the impact of these tools on mitigating distress.

**Objective:**

This study seeks to determine the acceptability, feasibility, usability, and effectiveness of mobile mental health apps for decreasing mental health symptoms in essential workers and unemployed individuals with suicide risk.

**Methods:**

We recruited participants who indicated that they were unemployed because of COVID-19 or were COVID-19–designated essential workers. Participants were randomized to 1 of 4 free commercial mobile apps for managing distress that were (1) highly rated by PsyberGuide and (2) met the criteria for intervention features these participants indicated were desirable in a previous survey. Participants used the apps for 4 weeks and completed baseline and 4-week self-assessments of depression, anxiety emotional regulation, and suicide risk.

**Results:**

We found no differences between the apps in any outcome but did find significant changes in depression and anxiety over time (Patient Health Questionnaire [PHQ]-9: estimate=–1.5, SE 0.2, 95% CI –1.1 to –1.8, *P*<.001; Generalized Anxiety Disorder Scale [GAD]-7: estimate=–1.3, SE 0.2, 95% CI –1.0 to –1.6, *P*<.001). We found no significant changes in suicidal behavior (Suicide Behaviors Questionnaire-Revised [SBQ-R]) or emotional regulation (Difficulties in Emotion Regulation Scale – Short Form [DERS-SF]) for the 4 weeks. We did find a significant dose-response pattern for changes in depression and anxiety. Using the app at least once a week resulted in greater improvements in treatment conditions over time on depression (estimate=–0.6, SE 0.2, 95% CI 1.0-0.2, *P*=.003) and anxiety (estimate=0.1, SE 0.2, 95% CI 0.4-0.6, *P*=.78). There was no association between app frequency and changes in suicidal behavior (SBQ-R) or emotional regulation (DERS-SF). We further found a significant difference between the conditions with regard to app usability, with the control app being the most usable (mean_Beautiful Mood_ 72.9, SD 16.7; mean_COVID Coach_ 71.2, SD 15.4; mean_Calm_ 66.8, SD 17.3; mean_7 Cups_ 65.2, SD 17.7). We found no significant differences for app acceptability or appropriateness.

**Conclusions:**

Few studies have evaluated prospectively the utility and usability of commercial apps for mood. This study found that free, self-guided commercial mobile mental health apps are seen as usable, but no one app is superior to the other. Although we found that regular use is indicated for effects on depression and anxiety to occur in those who are more symptomatic, regression to the mean cannot be ruled out.

**Trial Registration:**

ClinicalTrials.gov NCT04536935; https://tinyurl.com/mr36zx3s

## Introduction

### Background

Access to mental health care by essential workers and the people unemployed due to COVID-19–related business closures and social distancing policies has been challenging [1-3]. To address this problem, health care organizations have created free mobile apps for stress related to COVID-19. Although overall app use during COVID-19 has been low (16%) [4,5], technology companies report substantial increases in the use of their tools.

There is limited information about the effectiveness of mental health apps, particularly free, self-guided commercial apps. Research on self-guided apps is mixed, with some studies finding them to be minimally effective [6-8] and others reporting beneficial effects; we note here that most evidence points to the superiority of coach-based apps for depression and anxiety outcomes, but effect sizes for self-guided apps are still notable [9] and offer an opportunity for stress management in populations that do not have the financial or time resources to avail themselves of coaching services [10]. It is important to also note here that most studies that find positive effects use research grade tools with a paid participant pool and are typically not available to the public. Many commercial apps do include principals and features that are similar to research grade tools; however, there remains skepticism about the effectiveness of these derivations [11]. This has led to the need to create app review resources, such as One Mind PsyberGuide [12] and the American Psychiatry Association’s App Advisor [13], which provide ratings of app effectiveness, transparency, and usability. Still, evidence for free commercial apps is limited, and calls for additional research [14,15], particularly in the context of COVID-19 [16], have been made.

### Objective

We previously reported on a large-scale survey of essential workers and people unemployed due to COVID-19 for their preferences for mobile apps for mood management [4]. In this study, we found that participants had strong preferences for apps that focus on mindfulness approaches, information about coping with COVID-19, symptom tracking, and connection with others. In this pragmatic clinical trial, we randomized 838 of these participants who indicated they were depressed or anxious or had suicidal thoughts in order to use 1 of 4 commercial apps for 4 weeks. We selected the 4-week time frame because in our past research, we found that this is an optimal dose of digital mental health in a distressed sample [17] and other research has found that this is the length of time participants tend to engage with these tools [18,19]. Thus, we are interested in addressing the issues of app use and outcomes pragmatically, as it would occur in actual practice. The main objectives of this study were:

- Determine whether users of these apps show significant improvement in anxiety, depression, emotion regulation, and suicide risk.
- Identify differences between the apps in use, usability, and acceptability.
- Determine whether there is a dose-response relationship such that the frequency of app use is positively associated with improvement in depression, anxiety, emotional regulation, and suicide risk.
- Identify outcome differences between apps in this dose-response relationship.

## Methods

### Recruitment and Safeguards Against Bad Actors

Participants were recruited nationally via Prolific, an online research platform that includes several safeguards to preserve data quality [20-22] and minimize bad actors and has been shown to be reliable, efficient, and affordable for remote data collection for behavioral research [23]. Participants provided electronic informed consent prior to study completion. Additional survey safeguards were an attention check [24] and a review of open-ended items to screen out autofilled and nonsensical responses.

### Ethical Approval

The study received ethical approval from the University of Washington Institutional Review Board (STUDY00010842). In the consent, participants were explained the purpose of the study, that it would be randomized to 1 of 4 mobile apps, and that they would be asked to complete surveys before treatment began and 4 weeks later. Participants were also told how data were stored and managed and approximately how long each survey would take.

### Participants

Participants for this study were recruited from a larger study [4], which included a convenience sample of approximately 2000 participants that self-identified as COVID-19–designated essential workers or unemployed due to COVID-19 social distancing policies or COVID-19–related business closures. To identify as 1 of these 2 groups, participants responded to the following 2 questions: (1) Are you considered an essential worker during the COVID-19 pandemic? (2) Have you become unemployed as a result of the COVID-19 pandemic?

To be eligible for this study, inclusion criteria included (1) previously granting permission to be recontacted for future research; (2) age≥19 years, living in the United States, and English speaking; (3) access to a mobile device; and (4) report of depression (Patient Health Questionnaire [PHQ]-2 score≥3) [25], anxiety (Generalized Anxiety Disorder Scale [GAD]-2 score≥3) [26], risk for suicidal behaviors (Suicide Behaviors Questionnaire-Revised [SBQ-R] score≥7) [27], or a history of past suicide attempt [28]. Participants were offered crisis management resources when they endorsed the ninth item of the PHQ-9 or were over the cut-off for the SBQ-R.

### Study Timeline

Participants were recruited from October through December 2020, during the middle of the initial COVID-19 variant, and shortly after vaccines were available to the public. Additionally, most states (with few exceptions) were continuing to institute public closures of restaurants, gyms, and other enclosed public places, meaning the unemployment rate due to COVID-19 was still quite high. Hospital censuses were at historically high rates, and essential workers were still mandated to wear protective gear. Thus, the sample is representative of people living under peak pandemic conditions. Participants were randomized after completing a web-based baseline assessment of mood and paid US $1 (see the Measures section). Participants were randomized to 1 of 4 apps and asked to use their assigned app as instructed by the developers. Participants completed a web-based posttreatment survey at 4 weeks postrandomization and app assignment. After completing follow-up, participants were compensated US $4.

### Mobile Interventions and Attention Control

This remote pragmatic clinical trial used simple randomization with parallel assignment comparing 3 active apps to an attention control app. This study meets the definition of a pragmatic trial in that the study was designed to test the effects of mobile apps for depression, anxiety, emotion regulation, and suicide as they are typically used by the general public [29]. In pragmatic trials, the intent is to determine the effect of existing treatments in the context of real-world use compared to existing treatment options. In such trials, the control condition is not a placebo, which is not usually part of standard care [30]. Although a waitlist may be appropriate for a pragmatic trial, waitlist controls are appropriate only when this is part of usual practice and if they are ethically sound; however, previous research has found internal validity issues with waitlist controls, and in the context of self-guided commercial digital mental health, there is no waitlist control [31]. In our sample, which consisted of participants at risk for suicide, neither a placebo nor waitlist controls were ethical choices [32]. Thus, our decision to use an attention control app was based on what is considered appropriate for pragmatic trials of this nature in potentially high-risk populations [33,34].

We selected apps based on the following criteria: (1) they were free; (2) reflected desired app features during COVID-19, as identified in the survey study [4]; and (3) had good ratings on PsyberGuide [4]. The 3 active app interventions included (1) meditation (Calm), (2) COVID-19 coping (COVID Coach), and (3) chat and positive psychology (7 Cups of Tea). The attention control app used only mood tracking (Beautiful Mood) and did not include any intervention elements the other apps possessed (mindfulness meditation, emotional coping skills, social connection, or positive psychology approaches). Participants were randomized by study staff using random allocation functions in Microsoft Excel and received their app assignment through a URL to Google Play Store or Apple App Store. Participants confirmed app download prior to receiving compensation. Participants were blinded to the study hypotheses but not condition.

### Rationale for the 4-Week Intervention Timeline

We feel it is important to note that although mental health apps are based on evidence-based treatment approaches, people use apps differently than the way they use traditional mental health services [35]. The optimal dose of mobile mental health apps is measured in the frequency of use rather than the number of weeks of use, and research shows that considerable improvement in mood and function can occur rapidly with digital mental health tools and as early as after 2 weeks of use [17,36,37]. We acknowledge that although other randomized clinical studies do show the greatest impact at 8 weeks [38], the general population tends to initially engage with digital mental health apps frequently over the course of 2 weeks, with notable disengagement by 4 weeks [11,18,19]. Based on the literature from the informatics field on typical engagement patterns with digital health tools in general, this is a common pattern of engagement and may mean the user has met their goal [18].

### Measures

All data collected for this study are considered sensitive. We did not collect or store names, addresses, locations, IP addresses, or other digital identifiers. All survey data, including demographics, were immediately stored behind secure firewalls on servers at the University of Washington School of Medicine. The survey was developed by the study’s lead investigators (authors PAA and KAM), measures were selected for their validity and reliability, and we selected those measures that had been validated for online use. The survey was programmed into REDCap, a web-based survey program developed by Vanderbilt University [39]. It has been used extensively for clinical research and is Health Insurance Portability and Accountability Act (HIPAA) compliant, highly secure, and intuitive to use. After the survey was built, we tested it with research group members naive to the study for readability, programming bugs, and time to completion.

#### Demographics

Participants provided information about age, race and ethnicity, gender identity, sexual orientation, education, income, and living situation. We used similar questions to those in the US Census categories [40]. This survey has been used successfully in other online studies [4,41]. See Multimedia Appendix 1. Race, ethnicity, and gender were assessed because mental health disparities were present in these groups [42,43].

#### Primary Clinical Outcomes

Participants completed measures of depression (PHQ-9) [25,44,45], anxiety (GAD-7) [26,46,47], emotion dysregulation (Difficulties in Emotion Regulation – Short Form [DERS-SF]) [48], and suicidal behaviors (SBQ-R) [27] at baseline and follow-up. The PHQ-9 score ranges from 0 to 27, with 0-4 indicating no depression, 5-9 indicating mild depression, 10-20 indicating moderate depression, and a score>20 indicating severe depression. The GAD-7 is scored from 0 to 21, with 0-4 indicating no anxiety, 5-9 indicating mild anxiety, 10-14 indicating moderate anxiety, and 15-21 indicating severe anxiety. The DER-SF is scored from 1 to 180 and, while showing strong psychometric properties in clinical populations, does not have a clinical cut-off. The SBQ-R is scored from 3 to 18 and has a nonclinical cut-off of 7 and a clinical cut-off of 8 for elevated suicide risk [49].

The PHQ-9 and GAD-7 have been used successfully as online survey instruments and have been validated as online instruments [50,51]. Although the DERS and SBQ-R have been used in online survey research [52,53], to the best of our knowledge, no formal tests of validity have been conducted. We still elected to use these scales as there is no existing validated instrument for emotion regulation and suicide behaviors and because, of the existing scales, these have the best psychometric properties, are valid and reliable across demographic groups, and are least burdensome to administer owing to a shorter length and ease of understanding [27,54-66].

#### App Use

As we were not able to collect in-app use data, participants were asked how often they used the app that they were assigned to over the past 4 weeks on a scale of 1 (*never downloaded the app*) to 8 (*multiple times per day*). To ease interpretation, results presented here are for response options collapsed into 4 categories, with findings highly similar in both categorization schemes. Categories included 1 (*never downloaded the app* and *downloaded but did not use the app*), 2 (*rarely* [*1-3 times in the past month*] and *infrequently* [*less than weekly*]), 3 (*weekly* and *more than weekly but less than daily*), and 4 (*daily* and *multiple times per day*).

#### Fidelity

A dichotomized fidelity measure was created in accordance with each app’s recommended use found on its website. Daily use was recommended for the apps Beautiful Mood [67], COVID Coach [68], and Calm [69], while weekly use was recommended for 7 Cups of Tea [70].

#### Usability

App usability was assessed with the Intervention Usability Scale (IUS) [71], a 10-item measure that assesses psychosocial intervention usability through its likeability, learnability, difficulty, need for support, system integration, and efficiency. This measure is based on the System Usability Scale [72], a standardized, normed measure in industry for digital tools, and has been validated for online research [73,74]. The IUS is scored from 0 to 100, and a score of 85 or more is considered to be excellent usability [75].

#### Acceptability and Appropriateness

The degree to which the app was satisfactory and appropriate (ie, the fit and relevance of the intervention) was measured with the Acceptability Intervention Measure (AIM) and the Intervention Appropriateness Measure (IAM) [76]. These scales’ scores range from 0 to 20, with higher scores indicating great acceptability (AIM) and appropriateness (IAM). Both measures contain 4 items that exhibit good psychometric properties, and the items have been validated by implementation scientists and mental health professionals. Although these measures have not been validated for online use, they are the only validated instruments for intervention acceptability and appropriateness, are brief, and face-valid [76].

### Survey Administration

The survey was completed by volunteers identified through our initial sample [4]. Participation in the study, including survey completion, was voluntary. After volunteering on Prolific and providing consent, participants completed all measures in a web-based REDCap interface. The survey included 14 measures, each on a separate page, with 4-18 questions per measure. Participants were able to review and change their responses on each measure before proceeding to the next measure. The survey items were not randomized, as each scale used must be delivered in the order it was validated. We did not use skip patterns or other survey logic; participants were asked to complete all survey questions and had the option to not answer certain questions.

### Statistical Methods

Prior to analyses, we examined the data and eliminated participants for not meeting inclusion or good-actor criteria. Good-actor criteria required participants to correctly answer an attention check item, answer at least 50% of the items on the survey, complete the survey faster than 33% of the median length of time, and not have any problems with the Prolific ID (not being an approved ID, being a duplicate ID, or having a missing ID). We performed *t* tests and cross-tabulations with chi-square tests to compare demographic and baseline clinical outcomes by missing data status at the follow-up time point. Chi-square tests examined the association between condition assignment and compliance (whether a participant used the app they were assigned or used an alternative app). All analyses were of the intention-to-treat (ITT) type. Analyses of variance were used to compare conditions on AIM, IAM, and IUS scores at follow-up, with Tukey honestly significant difference (HSD) tests making pairwise post hoc comparisons among all apps. Mixed effects models using restricted maximum likelihood estimation were built to test the linear time change on the PHQ-9, GAD-7, SBQ-R, and DERS-SF and to test for condition differences at follow-up and change slope. We applied mixed effects models with 2 time points with within-person nesting. We used this rather than alternatives, such as a simple regression, because mixed effects models efficiently (1) simplified simultaneous testing of within-person changes via testing slope coefficients and between-condition differences via testing condition coefficients; (2) facilitated testing as we built models progressively adding time trends, condition effects, and dosage effects; and (3) permitted the inclusion of random intercepts to account for variance at baseline, which was particularly important for testing models of dosage. Models were built and tested in an outwardly nested fashion, such that an initial null model was computed, followed by models that added a random intercept, random time component, condition assignment with Beautiful Mood as the reference variable (the attention control condition), and condition × time interaction effects. To test whether there was a dose-response relationship such that the app use frequency was associated with the rate of change on the PHQ-9, GAD-7, SBQ-R, and DERS-SF scores, we computed another series of mixed effects models for each outcome. An initial model included variables for time and frequency of use, a second model added condition terms for each app using Beautiful Mood as the reference, a third model added condition × frequency interaction terms for each app, and a fourth model included time, frequency, and a time × frequency interaction term. Model comparisons applied –2 log likelihood (–2LL), the Akaike information criterion (AIC), and Bayesian information criterion (BIC) deviance statistics. To test the impact of app use frequency on change over time, similar nested model testing was applied using an initial null model, followed by models that added dosage, dosage × time interaction, and condition assignment.

#### Sample Size and Power

A priori power analysis for an ANOVA *F* test indicated that a sample size of 800 (n=200 participants in each of the active and control app conditions) would be sufficient with power=0.80 and α=.05 for a minimum detectable effect size (MDES) of Cohen d=0.24 for main effect comparisons between any 2 conditions. The post hoc power analysis for 643 participants with complete data found an MDES of Cohen d=0.29 for main effect comparisons between any 2 conditions. Previous research has found an average Hedges g effect size (a comparable effect size to Cohen d but corrected for small samples) on self-guided mental health apps to be 0.50 and for self-guided tools to be 0.24 [77].

## Results

### Recruitment

**Figure 1:**
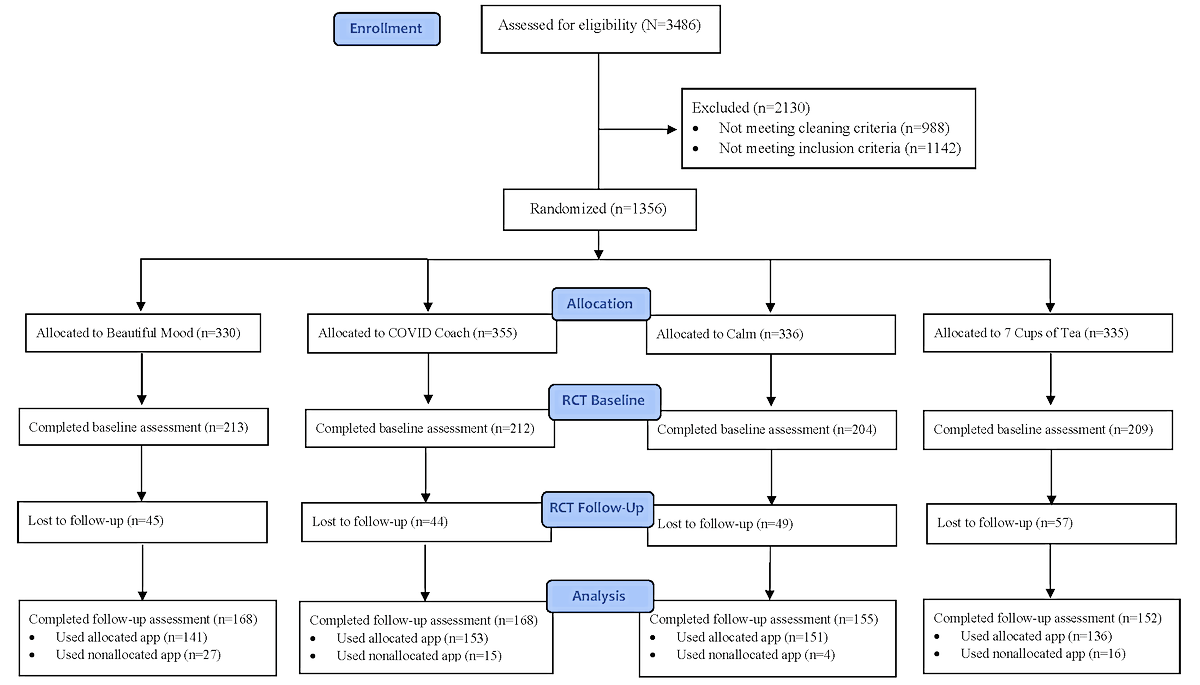
Consolidated Standards of Reporting Trails (CONSORT) table.

Figure 1 presents the Consolidated Standards of Reporting Trails (CONSORT) diagram. There were 3486 individuals assessed for eligibility, 2130 (61.1%) were excluded for not meeting good-actor criteria (n=988, 46.4%) or not meeting randomized clinical trial (RCT) inclusion criteria (n=1142, 53.6%). A total of 1356 (38.9%) individuals were randomized to Beautiful Mood (n=330, 24.3%), COVID Coach (n=355, 26.2%), Calm (n=336, 24.8%), or 7 Cups of Tea (n=335, 24.7%). Among those allocated to a condition, 838 (61.8%) participants completed the RCT baseline assessment, while 643 (47.4%) participants completed the follow-up assessment. In addition, 581 (90.4%) participants reported using the assigned app and 62 (9.6%) reported using a nonassigned app. For this ITT trial, all randomized participants were included in the primary analysis.

### Sample Description

Table 1 presents individual-level demographic data. The analytic sample consisted of 838 adults, with a mean age of 31.1 (SD 9.5) years. Most patients identified as women (467/833, 56.1%) and White (616/838, 73.5%). Participants self-identified as unemployed due to COVID-19 (428/838, 51.1%) or COVID-19–designated essential workers (410/838, 48.9%). There were no significant differences in demographics by condition.

**Table 1:** Sample descriptive statistics for 4 conditions using different self-guided mobile apps.

Demographics			Beautiful Mood (n=213)		COVID Coach (n=212)		Calm (n=204)		7 Cups of Tea (n=209)		Total (N=838)
**Race, n (%), *P*=.28^a^**											
	African American/Black	15 (7.0)		20 (9.4)		11 (5.4)		12 (5.7)		58 (6.9)	
	American Indian/Alaska Native	3 (1.4)		2 (0.9)		3 (1.5)		0		8 (1.0)	
	Asian	22 (10.3)		23 (10.8)		26 (12.7)		13 (6.2)		84 (10.0)	
	Multiracial	13 (6.1)		18 (8.5)		10 (4.9)		12 (5.7)		53 (6.3)	
	Other race^b^	4 (1.9)		7 (3.3)		5 (2.5)		3 (1.4)		19 (2.3)	
	White	156 (73.2)		142 (67.0)		149 (73.0)		169 (80.9)		616 (73.5)	
**Ethnicity, n (%), *P*=.79^a^**											
	Hispanic/Latinx	23 (10.9)		17 (8.2)		21 (10.3)		19 (9.1)		80 (9.6)	
	Not Hispanic/Latinx	188 (89.1)		190 (91.8)		183 (89.7)		189 (90.9)		750 (90.4)	
	Missing	2 (0.9)		5 (2.4)		0		1 (0.5)		8 (1.0)	
**Age (years), *P*=.16^c^**											
	Mean (SD)	31.8 (10.1)		29.9 (8.2)		31.4 (10.0)		31.4 (9.4)^d^		31.1 (9.5)^e^	
**Gender, n (%), *P*=.86^a^**											
	Women	116 (54.7)		118 (56.5)		118 (57.8)		115 (55.3)		467 (56.1)	
	Gender diverse	0		1 (0.5)		1 (0.5)		2 (1.0)		4 (0.5)	
	Men	85 (40.1)		80 (38.3)		78 (38.2)		83 (39.9)		326 (39.1)	
	Nonbinary	10 (4.7)		7 (3.3)		7 (3.4)		7 (3.4)		31 (3.7)	
	Transgender	1 (0.5)		3 (1.4)		0		1 (0.5)		5 (0.6)	
	Missing	1 (0.5)		3 (1.4)		0		1 (0.5)		5 (0.6)	
**Marital status, n (%), *P*=.41^a^**											
	Divorced	14 (6.7)		11 (5.3)		10 (4.9)		17 (8.2)		52 (6.3)	
	Married (including same-sex partnership)	61 (29.0)		56 (26.8)		56 (27.6)		61 (29.5)		234 (28.2)	
	Never married	134 (63.8)		136 (65.1)		131 (64.5)		123 (59.4)		524 (63.2)	
	Separated	1 (0.5)		6 (2.9)		4 (2.0)		3 (1.4)		14 (1.7)	
	Widowed	0		0		2 (1.0)		3 (1.4)		5 (0.6)	
	Missing	3 (1.4)		3 (1.4)		1 (0.5)		2 (1.0)		9 (1.1)	
**Education, n (%), *P*=.47^a^**											
	High school, General Educational Development (GED), or less	22 (10.3)		28 (13.3)		27 (13.2)		23 (11.0)		100 (11.9)	
	Some college	74 (34.7)		50 (23.7)		54 (26.5)		51 (24.4)		229 (27.4)	
	Trade/technical/vocational	19 (8.9)		27 (12.8)		25 (12.3)		32 (15.3)		103 (12.3)	
	Bachelor's degree	64 (30.0)		73 (34.6)		65 (31.9)		67 (32.1)		269 (32.1)	
	Higher education	34 (16.0)		33 (15.6)		33 (16.2)		36 (17.2)		136 (16.2)	
	Missing	0		1 (0.5)		0		0		1 (0.1)	
**Income (US $), n (%), *P*=.28^a^**											
	<10,000	30 (14.2)		37 (18.0)		31 (15.3)		30 (14.5)		128 (15.5)	
	10,000-31,199	60 (28.3)		56 (27.2)		58 (28.6)		53 (25.6)		227 (27.4)	
	31,200-33,280	17 (8.0)		17 (8.3)		5 (2.5)		8 (3.9)		47 (5.7)	
	33,281-49,999	29 (13.7)		31 (15.0)		31 (15.3)		31 (15.0)		122 (14.7)	
	50,000-59,999	11 (5.2)		16 (7.8)		23 (11.3)		17 (8.2)		67 (8.1)	
	60,000-69,999	10 (4.7)		10 (4.9)		14 (6.9)		14 (6.8)		48 (5.8)	
	70,000-99,999	34 (16.0)		19 (9.2)		20 (9.9)		27 (13.0)		100 (12.1)	
	100,000-149,999	14 (6.6)		15 (7.3)		11 (5.4)		14 (6.8)		54 (6.5)	
	≥150,000	7 (3.3)		5 (2.4)		10 (4.9)		13 (6.3)		35 (4.2)	
	Missing	1 (0.5)		6 (2.8)		1 (0.5)		2 (1.0)		10 (1.2)	
**Employment, n (%), *P*=.47^a^**											
	Essential worker	112 (52.6)		107 (50.5)		95 (46.6)		96 (45.9)		410 (48.9)	
	Unemployed	101 (47.4)		105 (49.5)		109 (53.4)		113 (54.1)		428 (51.1)	

^a^Chi-square *P* value.

^b^Other race: most common responses for race were Hispanic, Mexican, and mixed.

^c^ANOVA *F* test *P* value.

^d^N=208.

^e^N=837.

### Missing Data

A total of 643/838 (76.7%) participants completed the follow-up assessment. There were no significant differences between those missing or not missing the follow-up assessment in the demographic data in Table 1 or clinical measures at baseline.

### Randomization Adherence and Compliance

At follow-up, 62/643 (9.6%) participants reported being nonadherent to condition assignment and reported that they use a different app than the one they were randomly assigned to use. Participants who were randomized to Beautiful Mood were less likely to use their assigned app, while individuals randomized to Calm were more likely to use their assigned app (*P*<.001).

### App Use

A cross-tabulation with the chi-square test found significant differences between the apps in the amount of use the participants reported; participants used Beautiful Mood more frequently and COVID Coach and 7 Cups of Tea less frequently (see Table 2).

**Table 2:** App compliance and use frequency^a^ by condition (*P*<.001^b^).

App use and compliance		Beautiful Mood (n=168), n (%)	COVID Coach (n=168), n (%)	Calm (n=155), n (%)	7 Cups of Tea (n=152), n (%)	Total (N=643), n (%)	
Adherent to app assignment		141(83.9)	153 (91.1)	151 (97.4)	136 (89.5)	581 (90.4)	
**App use**							
	Never downloaded/no use	18 (10.7)	14 (8.3)	15 (9.7)	21 (13.8)	68 (10.5)	
	Rarely/infrequently	*45 (26.8)* ^c^	*80 (47.6)* ^c^	62 (40.0)	*72 (47.4)* ^c^	259 (40.3)	
	Weekly or more	56 (33.3)	55 (32.7)	55 (35.5)	52 (34.2)	218 (33.9)	
	Daily/multiple times per day	*49 (29.2)* ^c^	19 (11.3)	23 (14.8)	*7 (4.6)* ^c^	98 (15.2)	

^a^According to their websites, Beautiful Mood, COVID Coach, and Calm apps recommend daily use, while 7 Cups of Tea recommends weekly use.

^b^Chi-square *P* value.

^c^Italicized values indicate a significant difference indicated by standardized residuals.

### Usability, Acceptability, and Appropriateness

ANOVA found a significant difference on the IUS between the conditions (mean_Beautiful Mood_ 72.9, SD 16.7; mean_COVID Coach_ 71.2, SD 15.4; mean_Calm_ 66.8, SD 17.3; mean_7 Cups_ 65.2, SD 17.7). Tukey HSD post hoc tests indicated Beautiful Mood is significantly more usable than Calm (mean difference 6.0, 95% CI 1.2-10.8, *P=*.01) and 7 Cups of Tea (mean difference 7.7, 95% CI 2.9-2.5, *P*<.001). COVID Coach was significantly more usable than 7 Cups of Tea (mean difference 6.1, 95% CI 1.2-10.9, *P=*.01). We found no significant differences in app acceptability (overall AIM mean 3.5, SD 1.0, 95% CI 3.4-3.6, *P*=.22) or appropriateness (overall IAM mean 3.6, SD 0.9, 95% CI 3.6-3.7, *P*=.48).

### Clinical Outcomes

Table 3 displays the reporting sample size, mean scores, and SDs at each time point for the PHQ-9, GAD-7, SBQ-R, and DERS-SF for each app.

**Table 3:** Pretest and posttest scores on clinical outcomes by condition.

App and time		PHQ^a^-9			GAD^b^-7			SBQ-R^c^			DERS-SF^d^	
		n (%)	Mean (SD)	n (%)		Mean (SD)	n (%)		Mean (SD)	n (%)		Mean (SD)
**Beautiful Mood (n=168)**												
	Pretest	165 (98.2)	10.6 (6.6)	167 (99.4)		8.8 (5.7)	154 (91.7)		7.1 (3.6)	165 (98.2)		44.5 (13.0)
	Posttest	165 (98.2)	9.1 (6.5)	167 (99.4)		7.8 (5.6)	154 (91.7)		7.0 (3.8)	165 (98.2)		44.6 (14.2)
**COVID Coach (n=168)**												
	Pretest	166 (98.8)	11.2 (6.3)	168 (100)		9.2 (5.6)	159 (94.6)		6.9 (3.8)	163 (97.0)		44.6 (12.5)
	Posttest	166 (98.8)	9.8 (6.7)	168 (100)		7.8 (5.6)	159 (94.6)		6.7 (3.7)	163 (97.0)		44.6 (14.0)
**Calm (n=155)**												
	Pretest	155 (100)	10.1 (5.7)	153 (98.7)		7.9 (4.8)	144 (92.9)		6.6 (3.3)	153 (98.7)		42.9 (11.7)
	Posttest	155 (100)	8.5 (5.9)	153 (98.7)		6.7 (5.1)	144 (92.9)		6.4 (3.3)	153 (98.7)		42.2 (13.1)
**7 Cups of Tea (n=152)**												
	Pretest	151 (93.3)	11.0 (6.5)	151 (93.3)		8.9 (5.6)	139 (91.4)		7.1 (3.9)	148 (97.4)		44.6 (12.8)
	Posttest	151 (93.3)	9.7 (6.6)	151 (93.3)		7.3 (5.8)	139 (91.4)		7.1 (3.9)	148 (97.4)		44.9 (13.0)

^a^PHQ: Patient Health Questionnaire.

^b^GAD: Generalized Anxiety Disorder Scale.

^c^SBQ-R: Suicide Behaviors Questionnaire-Revised.

^d^DERS-SF: Difficulties in Emotion Regulation Scale – Short Form.

Examining the –2LL, AIC, and BIC deviance statistics for each of the 4 analyses revealed that the best-fitting model was a random intercept model with a linear time slope in 2 cases: the PHQ-9 (–2LL=9118, dev_1_=63.2, *P*<.001; AIC=9128, dev_1_=61.2, *P*<.001; BIC=9137, dev_1_=71, *P*<.001; parameters=5) and GAD-7 (–2LL=8686, dev_1_=71.7, *P*<.001; AIC=8696, dev_1_=69.7, *P*<.001; BIC=8720, dev_1_=64.9, *P*<.001; parameters=5). From baseline to follow-up, participants improved by an estimated –1.5 points on the PHQ-9 (SE 0.2, 95% CI –1.1 to –1.8, *P*<.001) and –1.3 points on the GAD-7 (SE 0.2, 95% CI –1.0 to –1.6, *P*<.001). Models that included condition main effects centered at the follow-up time point and condition × time were not significantly better fitting than the random intercept and time model. For the other 2 analyses, the SBQ-R and DERS-SF, the best-fitting models were the null models, with no random terms or interaction variables (SBQ-R: –2LL=6695, AIC=6703, BIC=6722, parameters=4; DERS-SF: –2LL=11,078, AIC=11,086, BIC=11,105, parameters=4). Thus, there were significant mean improvements in the PHQ-9 and GAD-7 scores but not the SBQ-R and DERS-SF scores, and the app condition was not associated with differences in any of the 4 analyses.

### Dosage

Mixed effects models were computed to examine the relation of frequency of app use with change over time on the PHQ-9, GAD-7, SBQ-R, and DERS-SF, over all conditions, controlling for the condition, for a condition × app use interaction, and for condition × time. The best-fitting model for the PHQ-9 included time, frequency of app use, and frequency × time interaction, as indicated by 2 of the 3 fit indices (–2LL=7868, dev_1_=8.9, *P*=.002; AIC=7882, dev_1_=6.9, *P*=.01; BIC=7914, dev_1_=2.4, *P*=.12; parameters=7, *P*<.001). The BIC statistic, which penalizes for model complexity, was not statistically significant; therefore, these results should be viewed with some caution. All 3 fit statistics indicated that this same model structure was the best fit for the GAD-7 (–2LL=7481, dev_1_=14.2, *P*<.001; AIC=7495, dev_1_=12.2, *P*<.001; BIC=7527, dev_1_=7.8, *P*=.01; parameters=7). None of the models that included condition or condition × frequency of app use was a significantly better fit, meaning we found no differences between the treatment groups on the impact that app use frequency had on change over time. For the SBQ-R and DERS-SF, none of the more complex models improved on the fit of the initial model that included the time and frequency of app use.

**Figure 2:**
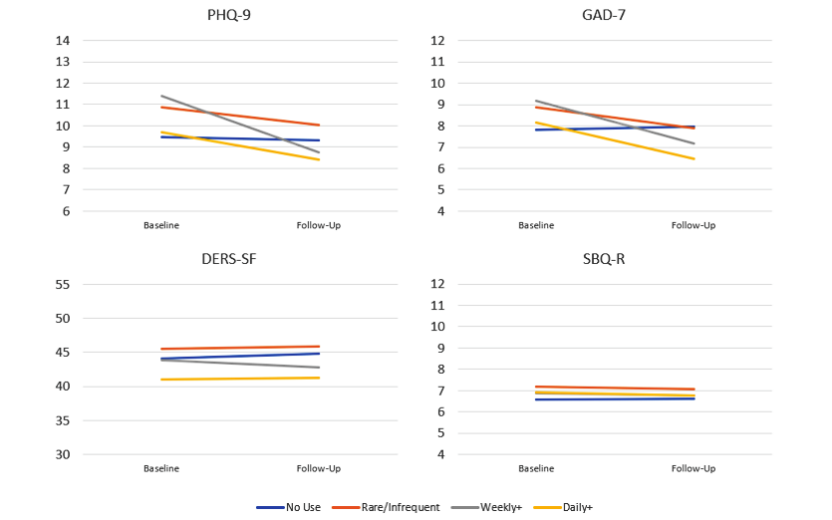
Clinical outcome means. DERS-SF: Difficulties in Emotion Regulation Scale – Short Form; GAD: Generalized Anxiety Disorder Scale; PHQ: Patient Health Questionnaire; SBQ-R: Suicide Behaviors Questionnaire-Revised.

Salient model parameters for the best-fitting models were as follows. For the PHQ-9, when time, frequency of app use, and time × frequency interaction were included in the best-fitting model, the frequency of app use was not significant (estimate=0.05, SE 0.3, 95% CI 0.5-0.6, *P*=.86) and time was not significant (estimate=0.1, SE 0.6, 95% CI 1.0-1.3, *P*=.80), but the time × frequency interaction was significant (estimate=–0.6, SE 0.2, 95% CI 1.0 to –0.2, *P*=.003). There were similar findings for the GAD-7 such that when time, frequency of app use, and time × frequency interaction were included in the best-fitting model, the frequency of app use was not significant (estimate=0.1, SE 0.3, 95% CI 0.4-0.6, *P*=.78) and time was not significant (estimate=0.4, SE 0.5, 95% CI 0.6-1.3, *P*=.43), but the time × frequency interaction was significant (estimate=–0.7, SE –0.7, 95% CI 1.0 to –0.3, *P*<.001). Figure 2 depicts the actual mean score for each condition by frequency. For the PHQ-9 and GAD-7, those who did not use the app had no significant change on that measure over time; those who used the app more frequently improved more quickly than those who used the app less frequently. By the 4-week follow-up, however, there were no significant differences in outcome by frequency of app use (dose).

For the SBQ-R, the best-fitting model indicated that the frequency of app use was not associated with lower scores overall (estimate=–0.03, SE 0.2, 95% CI 0.4-0.3, *P*=.16), although time was significant (estimate=–0.1, SE 0.1, 95% CI 0.3 to –0.003, *P*=.05); interaction terms were not included. Therefore, when statistically controlling for the frequency of app use, SBQ-R scores decreased over time but there was no association between app frequency and change on the SBQ-R.

For the DERS-SF, the best-fitting model indicated that the frequency of app use was associated with lower scores overall (estimate=–1.5, SE 0.6, 95% CI 2.6 to –0.4, *P*=.01), but time was not significant (estimate=–0.1, SE 0.3, 95% CI 0.8 to –0.6, *P*=.84), and interaction terms were not included. Those who used their app frequently had lower scores on the DERS-SF at baseline and follow-up, with no change on the DERS-SF over time and no association between frequency of app use and change on the DERS-SF.

## Discussion

### Principal Findings

To the best of our knowledge, this is 1 of the first pragmatic trials of free commercial apps among essential workers or those unemployed due to COVID-19 experiencing emotional distress and suicide risk. Our primary findings were that commercial mobile mental health apps are found to be usable and acceptable and have a positive impact on depression and anxiety but not emotional regulation or suicide risk. Although we did not find any significant difference between the 3 active apps on outcomes, nor between the active apps compared to the control app, we did find that the frequency of app use during the 4 weeks had a significant and positive impact on depression and anxiety outcomes. However, we offer here that 4 weeks of engagement may not be sufficient to show changes in emotional regulation or suicidal behavior or that online interventions may not be potent enough to manage these mental health challenges, given that those with greater emotion dysregulation used the apps less throughout the period. Indeed, a recent study offering online interventions aimed at suicide prevention not only found no effects but also demonstrated more adverse events in those offered online care versus those offered care as usual [78]. We also cannot rule out regression to the mean, as people who used the attention control app had outcomes similar to participants who used the active apps.

### Comparison With Previous Work

Our previous work suggests that apps that focus on mindfulness, pandemic information, mood tracking, and connection with others are an acceptable means of managing stress during COVID-19 [4]. Our findings on the lack of differential clinical impact between apps is not surprising, given the data from other pragmatic trials of research-grade mobile apps. In large-scale remote, pragmatic clinical trials of mobile apps for depression, all apps found significant improvements in mood over an 8-week period but no differences between groups [17]. Our findings regarding the importance of app use on clinical outcomes have also been found in previous studies on research-grade mobile apps, where frequent use of a mobile mood app early in care resulted in better depression outcomes for those who were more severely distressed [17,79]. Although smaller, controlled trials of self-guided apps in a research context do find small but statistically significant differences in outcomes compared to waitlist controls or no treatment [76], this study, and other pragmatic trials to date, have not demonstrated that active apps are more effective than attention control apps [7,78].

### Study Strengths and Limitations

The strength of this study is in its design: It is 1 of the first studies to evaluate free commercial mental health apps prospectively and independently in a large-scale, pragmatic RCT and to assess their impact on emotional distress, emotional regulation, and suicide risk in 2 suicide-vulnerable populations at the peak of the COVID-19 pandemic. The lessons learned from this study can be useful to people seeking free and readily accessible help for emotional distress. For the field, more work is needed to understand what role commercial apps can play for emotional distress, and the data from this study serve as a good starting point for understanding what is acceptable and effective and what optimal engagement should be.

Study limitations include the following:

- Our sample consisted of participants from Prolific and thus may be most representative of essential workers or those unemployed due to COVID-19 who are proactively seeking other sources of income to offset financial stress. Although this sample may be more comfortable with technology, we believe that people seeking mobile mental health apps are also comfortable with technology, and thus the results from this study are representative of this population.
- Because we did not partner with the technology companies who created the study apps, we relied on self-reported app use, which may be subject to self-report bias. However, incentives for participating in this study were not tied to app use, and data from numerous intervention studies find that people are highly accurate in their reports of intervention adherence [80-82].
- Although we justify our timeline for measuring outcomes after 4 weeks of intervention use based on what is typical for most mental health app users, we do not have information on the lasting effects of treatment outcomes or on continued app use. Thus, although we can report on the immediate effects of the intervention, future research is needed to determine the permanence of treatment effects.
- We did not ask about potential adverse events related to app use. This is an interesting area of research that to date has not been explored. Understanding the risks of using commercial apps is as important as determining their impacts and should be explored in future studies.

### Conclusion

There are several papers calling for more research to study the effectiveness of commercial mental health app interventions [7,10,83,84], specifically for COVID-19 [85-88], but published studies to date report only app downloads, aesthetics, and app use [6,83,89]. Our data suggest that essential workers and those unemployed who want self-guided mental health care found 4 commercially available apps both acceptable and usable and might receive emotional benefit from a variety of self-guided mental health apps, particularly if they use the apps frequently, but that regression to the mean cannot be ruled out, so improvement in symptoms may not be attributable to app use.

## Appendix

Multimedia Appendix 1 Demographics survey.

Multimedia Appendix 2 CONSORT-eHEALTH checklist (V 1.6.1).

## Abbreviations

–2LL: –2 log likelihood
AIC: Akaike information criterion
AIM: Acceptability Intervention Measure
BIC: Bayesian information criterion
DERS-SF: Difficulties in Emotion Regulation Scale – Short Form
GAD: Generalized Anxiety Disorder Scale
HSD: honestly significant difference
IAM: Intervention Appropriateness Measure
ITT: intention-to-treat
IUS: Intervention Usability Scale
MDES: minimum detectable effect size
PHQ: Patient Health Questionnaire
RCT: randomized clinical trial
SBQ-R: Suicide Behaviors Questionnaire-Revised

## Data Availability

The raw data supporting the conclusions of this paper will be made available by the authors, without undue reservation.
