# Supplementary material for: Effectiveness of Mental Health Apps for Distress During COVID-19 in US Unemployed and Essential Workers: Remote Pragmatic Randomized Clinical Trial
Source: JMIR Mhealth Uhealth. 2022 Nov 7;10(11):e41689. doi: 10.2196/41689 (PMC9642829; doi:10.2196/41689)
Supplement: Multimedia Appendix 1 [file mhealth_v10i11e41689_app1.docx]

**Multimedia Appendix 1. Demographics survey.**

1. What city do you live in? [write-in]
2. What state do you live in? [drop-down]
3. What is your zip code? [write-in]
4. What is your race? (check all that apply)
   1. Asian
   2. White
   3. African American/Black
   4. Hawaiian/Pacific Islander
   5. American Indian/Alaska Native
      1. Tribal affiliation (optional): [write-in]
   6. Other
      1. Please specify “other:” [write-in]
5. What is your ethnicity?
   1. Hispanic/Latinx
   2. Not Hispanic/Latinx
6. What is your age? [write-in]
7. To what gender identity do you most identify? [write-in]
8. Do you consider yourself to be:
   1. Heterosexual/straight
   2. Gay/lesbian/homosexual
   3. Bisexual
   4. Other. Describe: [write in]
   5. I cannot/do not want to answer
9. In the past 5 years, have you had consensual sex with:
   1. only females
   2. only males
   3. both males and females
   4. no consensual sex past 5 years
   5. I cannot/do not want to answer
10. People are different in their sexual attraction to other people. Which best describes your feelings? Are you…
    1. Only attracted to females
    2. Mostly attracted to females
    3. Equally attracted to females and males
    4. Mostly attracted to males
    5. Only attracted to males
    6. Other. Describe: [write-in]
    7. I cannot/do not want to answer
11. What is your current relationship status?
    1. Single (not dating anyone currently)
    2. Dating casually, not living with a partner
    3. In a monogamous relationship (including marriage or same sex partnership), living apart
    4. In a monogamous relationship (including marriage or same sex partnership), living together
    5. Other relationship status. Describe: [write-in]
    6. I cannot/do not want to answer
12. What is your current marital status?
    1. Never married
    2. Widowed
    3. Married (including same sex partnership)
    4. Separated
    5. Divorced
    6. I cannot/do not want to answer
13. How many children do you have? (include biological children, step-children, and foster children as makes sense to you): [write-in]
    1. -I cannot/do not want to answer
14. What is your highest level of education?
    1. No schooling completed
    2. 8^th^ grade
    3. Some high school, no diploma
    4. High school graduate or equivalent
    5. Some college, no degree
    6. Trade/technical/vocational training
    7. Associate degree
    8. Bachelor’s degree
    9. Master’s degree
    10. Professional or doctorate
15. Are you currently employed?
    1. Yes
    2. Not working and not receiving disability or retirement income, or unemployment
    3. Disabled/retired
    4. Receiving unemployment
    5. I cannot/do not want to answer
16. What is the typical number of hours you work per week?
    1. Less than 10 hours
    2. 10 - 19 hours
    3. 20 - 34 hours
    4. 35 - 40 hours
    5. Over 40 hours
    6. I cannot/do not want to answer
17. Please estimate your gross annual income (before taxes) for the last year based on routine/typical income sources (such as your or partner’s income, unemployment, or disability payments; don’t include one-time sources of income or subsidies for housing, food, etc.)
    1. Below $10K
    2. $10,000 - $31,199
    3. $31,200 – $33,280
    4. $33,281 – $49,999
    5. $50,000 – $59,999
    6. $60,000 – $69,999
    7. $70,000 – $99,999
    8. $100,000 – $149,999
    9. $150,000 - $199,999
    10. $200,000 – $249,999
    11. $250K and above
    12. I cannot/do not want to answer
18. What is your professional role? (e.g., social worker, high school student, etc.) [write-in]
19. What is your current living situation (apartment, house, clean and sober housing, shelter, etc.)?
    1. House
    2. Apartment
    3. Clean and sober housing
    4. Shelter
    5. Couch surfing
    6. Street homeless (car, street; no heat or running water)
    7. Other. Describe: [write-in]
    8. I cannot/do not want to answer
20. Who do you live with? Mark all that apply
    1. Spouse/romantic partner
    2. Children (your own or others’)
    3. Mother, stepmother, mother in law
    4. Father, stepfather, father in law
    5. Brother(s)
    6. Sister(s)
    7. Friend(s) or roommate(s)
    8. I live alone
    9. Other. List other people you live with: [write-in]
    10. I cannot/do not want to answer
